# Cardioprotective Effect of Paeonol on Chronic Heart Failure Induced by Doxorubicin *via* Regulating the miR-21-5p/S-Phase Kinase-Associated Protein 2 Axis

**DOI:** 10.3389/fcvm.2022.695004

**Published:** 2022-07-05

**Authors:** Cong Chen, Shuhong Liu, Gaozhen Cao, Yang Hu, Run Wang, Min Wu, Mingya Liu, Kai Hang Yiu

**Affiliations:** ^1^Division of Cardiology, Department of Medicine, The University of Hong Kong, Shenzhen Hospital, Shenzhen, China; ^2^Division of Cardiology, Department of Medicine, The University of Hong Kong, Queen Mary Hospital, Shenzhen, China

**Keywords:** paeonol, chronic heart failure, cardioprotective, miR-21-5p, SKP2

## Abstract

**Background:**

This study primarily explored the role of paeonol in doxorubicin (DOX)-induced chronic heart failure (CHF), considering the cardioprotective effect of paeonol on an epirubicin-induced cardiac injury.

**Methods:**

DOX-induced CHF-modeled rats were treated with paeonol. Cardiac function and myocardial damage in rats were evaluated by using the multifunction instrument, and the histopathology, apoptosis, and the expression of miR-21-5p and S-phase kinase-associated protein 2 (SKP2) in myocardium were detected. The target gene of miR-21-5p was confirmed by a dual-luciferase reporter assay. After the required transfection or paeonol treatment, the viability, apoptosis, mitochondrial membrane potential (MMP), and reactive oxygen species (ROS) of the DOX-induced cardiomyocytes were determined. Reverse-transcription quantitative-PCR (RT-qPCR) and Western blot were performed to quantify the expressions of miR-21-5p, SKP2, and apoptosis-related factors.

**Results:**

Paeonol improved cardiac function and also ameliorated the cardiac damage of CHF-modeled rats, where the downregulation of abnormally elevated myocardial damage markers, including brain natriuretic peptide, lactate dehydrogenase, renin, angiotensin II, aldosterone, and endothelin 1, was observed. Paeonol alleviated the histopathological injury and suppressed the apoptosis in CHF-modeled rats, inhibited miR-21-5p expression, and upregulated SKP2 expression *in vitro* and *in vivo*. miR-21-5p targeted SKP2. Paeonol and SKP2 increased the viability and MMP, but reduced apoptosis and ROS in the DOX-induced cardiomyocytes. miR-21-5p exerted effects opposite to PAE and SKP2, and it downregulated the expression of Bcl-2 and mitochondrion-Cytochrome c (Cyt c) and upregulated the expression of Bax, C-caspase-3, and cytoplasm-Cyt c. miR-21-5p reversed the effects of paeonol, and its effects were further reversed by SKP2.

**Conclusion:**

Paeonol shows a cardioprotective effect on DOX-induced CHF *via* regulating the miR-21-5p/SKP2 axis.

## Highlights

- Paeonol inhibited miR-21-5p expression in doxorubicin-induced chronic heart failure rats and in doxorubicin-induced cardiomyocytes.
- SKP2 was targeted by miR-21-5p and it was down-regulated in chronic heart failure rats and doxorubicin-induced cardiomyocytes.
- Paeonol showed cardioprotective effects on doxorubicin-induced chronic heart failure *via* regulating miR-21-5p/SKP2/PI3K/AKT axis.

## Introduction

Heart failure (HF) is a complex clinical syndrome resulting from abnormal structures or functions of the heart, which impairs the cardiac blood circulation and reduces the cardiac function to a level lower than the demands of metabolic needs of the body (1, 2). HF can be divided into chronic HF (CHF) and acute HF (AHF) (3, 4). Specifically, CHF is mostly caused by ischemic heart disease (5), while AHF usually occurs in the elderly and is mostly caused by acute severe arrhythmia, myocardial damage, or a sudden severity of heart disease, causing the failure of the originally normal or compensating heart during a short period of time or rapid deterioration of CHF (6). Therefore, as a progressive development process, CHF is the final stage of many cardiovascular diseases (2). Besides, antineoplastic chemotherapy can cause multiple forms of cardiotoxicity, including pericardial disease, arrhythmia, myocardial ischemia, and valvular dysfunction (7). Hence, it is of great significance to develop effective drugs for the prevention and treatment of CHF.

*Paeonia suffruticosa* has been used for the treatment of atherosclerotic cardiovascular disease (8, 9). Paeonol is an important active ingredient in the Chinese herbal medicine *Paeonia suffruticosa* (8, 10). With the extensive application of Moutan Cortex in clinical practice, scholars at home and abroad have conducted many studies on its active ingredients, including paeonol (11–13). Since then, the biological activities of paeonol, including anti-cancer, anti-inflammation, and anti-oxidation, have been gradually discovered (10, 14–16). A previous study has reported that paeonol could be rapidly absorbed into the bloodstream after a single intramuscular (i.m.) administration in rats, with the absolute bioavailability found to be 68.68% (17). Paeonol has currently been proven to have a cardioprotective effect on epirubicin-induced heart injury (18). Additionally, recent studies further demonstrated that paeonol pretreatment attenuates hypoxia-reoxygenation-induced injury of cardiomyocytes *via* the breast cancer type 1 sensitive protein (BRCA1)-dependent pathway, and paeonol attenuates transverse aortic constriction-induced HF through the ERK1/2 signaling pathway (19, 20). Also, paeonol suppresses vasculogenesis *via* regulating vascular smooth muscle phenotypic switching, and it promotes Opa1-mediated mitochondrial fusion by activating the CK2α-Stat3 pathway in diabetic cardiomyopathy (21, 22). Moreover, paeonol protects against myocardial ischemia/reperfusion-induced injury by mediating apoptosis and autophagy cross-talk (23). Nevertheless, the mechanism underlying the cardioprotective effect of paeonol on CHF remains to be further explored.

MicroRNAs (miRNAs) are small non-coding RNAs with important functions in various physiological and pathological processes, including cardiac diseases (24). For example, miR-1 regulates the development of epirubicin-induced heart injury (18). miR-665 promotes HF by regulating the transcription of phosphatase and tensin homolog (25). Besides, the downregulation of miR-21 suppresses doxorubicin (DOX)-induced cardiac alterations (26). In addition to these discoveries, the anti-cancer effect of paeonol on liver cancer is realized by regulating miR-21-5p, a mature body of miR-21 (27). However, whether paeonol exerts an effect on CHF by regulating miR-21-5p is required to be investigated.

In the present study, we established a DOX-induced CHF rat model and cultured the DOX-induced cardiomyocytes to analyze the role of paeonol in CHF and explored whether miR-21-5p was involved in the treatment of CHF through paeonol.

## Materials and Methods

### Experimental Animals

The animal trials in this study were approved by the Ethics Committee of Experimental Animals of the University of Hong Kong Shenzhen Hospital (Z2020011901X) and were performed in the University of Hong Kong Shenzhen Hospital. In this work, all animal experiments were performed in accordance with the guidelines of the China Council on Animal Care and Use, and every effort was dedicated to minimizing the pain and discomfort of the animals.

In brief, 24 male Sprague-Dawley rats (weight: 200–250 g; age: 8–10 weeks) were obtained from Cavens (Changzhou, China), fed in the same animal feeding unit, and given a 12-h dark/12-h light cycle in an SPF-controlled environment.

### Chemicals and Diagnostic Kits

The following chemicals and diagnostic kits were used for the experiments: DOX (HY-15142, MedChemExpress, Princeton, NJ, United States), paeonol (HY-N0159, MedChemExpress, Princeton, NJ, United States), sodium pentobarbital (B005, Jiancheng Bioengineering Institute, Nanjing, China), ethyl carbamate solution (HY-B1207, MedChemExpress, Princeton, NJ, United States), brain natriuretic peptide (BNP) ELISA Kit (ml003039, mlbio, Shanghai, China), lactate dehydrogenase (LDH) Kit (ml092996, mlbio), renin (RE) ELISA Kit (ml059179, mlbio), angiotensin II (Ang-II) ELISA Kit (ml058803, mlbio), aldosterone (ALD) Kit (H188, Nanjing Jiancheng Bioengineering institute), endothelin 1 (ET-1) ELISA Kit (ab133030, Abcam, Cambridge, United Kingdom), xylene (X139941, Aladdin, Shanghai, China), 100% ethanol (E130059, Aladdin), paraffin (S25190, Yuanye, Shanghai), glass slide (C1-9646-11, ASONE, Tokyo, Japan), hematoxylin (HY-N0116, MedChemExpress), eosin (G1100, Solarbio, Beijing, China), transferase dUTP nick end labeling (TUNEL) staining kit (C1091, Beyotime, Shanghai, China), Proteinase K (M049338, MREDA, Beijing, China), washing buffer (P0106, Beyotime), blocking solution (P0100B, Beyotime), PBS (M059191, MRREDA), DMEM (A4192101, Gibco, Waltham, MA, United States), 10% FBS (16140071, Gibco), 0.1% Triton X-100 (P0096, Beyotime), goat serum (C0265, Beyotime), primary antibodies against S-phase kinase associated protein 2 (SKP2) (ab183039, Abcam, United Kingdom), secondary antibodies (ab150077, Abcam), lipofectamine 3000 transfection reagent (L3000015, Invitrogen, Waltham, MA, United States), pGL3-basic vectors (VT1554, YouBio, Hunan, China), commercial dual-luciferase reporter assay kit (ab228530, Abcam), TRIzol (R0016, Beyotime), isopropanol (H822173, Macklin, Shanghai, China), miRNA isolation regent (DP501, TianGEN, Beijing, China), EasyScript First-Strand cDNA Synthesis SuperMix (AE301-02, TransGen, Beijing, China), PerfectStart Green qPCR SuperMix (AQ601-01, TransGen), cell counting kit-8 (CCK-8) solution (C0040, Beyotime), Annexin V-FITC/Propidium iodide (PI) apoptosis detection kit (K2003, APExBIO, Houston, TX, United States), 2′,7′-dichlorodihydrofluorescein diacetate (H_2_DCFDA) staining buffer (KGAF018, KeyGen Biotech, Nanjing, China), tetramethylrhodamine methyl ester (TMRM) staining buffer (T5428, Sigma-Aldrich, St. Louis, MO, United States), NP-40 (P0013F, Beyotime), BCA protein assay kit (P0009, Beyotime), SDS-PAGE gels (P0052A, Beyotime), PVDF membranes (ISEQ00010, Millipore, MA, United States), SKP2 (1:2,500, 48 kD, ab68455, Abcam, Cambridge, United Kingdom), Bcl-2 (1:2,000, 26 kD, ab196495, Abcam), C-Caspase-3 (1:500, 17 kD, ab49822, Abcam), Bax (1:3,000, 21 kD, ab32503, Abcam), Cyt C (1:500, 12 kD, ab13575, Abcam), GAPDH (1:10,000, 36 kD, ab181602, Abcam), COXIV (1:500, 17 kD, ab16056, Abcam), secondary antibody against rabbit IgG (1:5,000, ab205718, Abcam), secondary antibody targeting mouse IgG (1:5,000, ab205719, Abcam), and ECL developer solution (P0019, Beyotime).

### Experimental Design

The animal experiment was performed based on the protocol described in previous publications (28, 29). In brief, after 5 days of adaptive breeding, the rats were randomly divided into the following four groups: the Sham group (*n* = 6), the DOX group (*n* = 6), the DOX + Paeonol (25 mg/kg) group (*n* = 6), and the DOX + Paeonol (50 mg/kg) group (*n* = 6). The rats in the Sham group received no treatment, while those in the DOX group, the DOX + Paeonol (25 mg/kg) group, and the DOX + Paeonol (50 mg/kg) group were first given an intraperitoneal injection of 2.5 mg/kg DOX (HY-15142, MedChemExpress, Princeton, NJ, United States) once a week for 6 weeks. Then, the rats in the DOX + Paeonol (25 mg/kg) group and the DOX + Paeonol (50 mg/kg) group were separately given the intraperitoneal injection of paeonol (HY-N0159, MedChemExpress) at the doses of 25 mg/kg and 50 mg/kg, respectively, once a day for 7 days.

On the last day of injection with paeonol, three rats (*n* = 3) from each group were randomly selected for subsequent testing experiments. Their cardiac function was evaluated. The rats were anesthetized with 2% sodium pentobarbital (50 mg/kg) (B005, Jiancheng Bioengineering Institute, Nanjing, China) and sacrificed by cervical dislocation, and their blood samples and heart tissues were harvested for later studies.

### Determination of Cardiac Function Parameters

The cardiac function of rats was evaluated following a previous publication (30). In brief, the rats (*n* = 3) were intraperitoneally anesthetized with 20% ethyl carbamate solution (HY-B1207, MedChemExpress). Then, the right common carotid artery of the rat was exposed and connected to an RM6240 series multichannel physiological signal acquisition apparatus (Chengdu Instrument Factory, Chengdu, China). Hemodynamic parameters, including left ventricular systolic pressure (LVSP), left ventricle end diastolic pressure (LVEDP), +left ventricle dp/dtmax (+dp/dtmax), −left ventricle dp/dtmax (−dp/dtmax), and heart rate (HR) were determined. The successful establishment of the CHF model was deemed when the values of +dp/dtmax were reduced to 50% of the original value.

### Estimation of Biomarkers

The rats were anesthetized with 2% sodium pentobarbital and sacrificed by cervical dislocation, and their blood samples were harvested for later detection. After the collection from the rats (*n* = 3), the blood samples were centrifuged for 15 min (4,000 × *g*) to obtain the serum. Then, the serum samples were further added to the Synergy H1 Hybrid Multi-Mode Reader (Bio-Tek, Winooski, VT, United States) to evaluate the concentration of some general blood biomarkers, including BNP, LDH, RE, Ang-II, ALD, and ET-1.

### Histopathology Study

In this work, xylene (X139941) was ordered from Aladdin (Shanghai, China). Then, the gradient ethanol (70%, 85%, 95%, and 100%) was prepared using 100% ethanol (E130059, Aladdin). After collecting the heart tissues of rats, the samples were fixed in 4% paraformaldehyde (C104190, Aladdin) for 24 h at room temperature, and then the tissues were dehydrated with gradient ethanol (70%, 85%, 95%, and 100%) for 3 min, respectively. The heart tissues of rats were harvested and then embedded into paraffin (S25190, Yuanye, Shanghai). Then, the embedded tissues were cut into the slices with a thickness of 4.5 μm and fixed on a glass slide (C1-9646-11, ASONE, Tokyo, Japan), followed by the deparaffinization and staining with hematoxylin (HY-N0116, MedChemExpress) for 10 min and with eosin (G1100, Solarbio, Beijing, China) for 1 min at room temperature in sequence. Finally, the images of the heart tissue were observed under an optical microscope (DM4M, Leica, Solms, Germany) at a magnification of ×200.

### Terminal Deoxynucleotidyl Transferase dUTP Nick End Labeling Staining

The TUNEL staining kit (C1091, Beyotime, Shanghai, China) was used in this study. After being incubated with 20 μg/ml of Proteinase K (M049338, MREDA, Beijing, China), the slices of heart tissue were sequentially immersed in washing buffer (P0106, Beyotime) at 20°C for 20 min (min) and blocked using blocking solution (P0100B, Beyotime) at room temperature for 20 min. Subsequently, the tissue slices were first incubated with 0.05 ml of marker buffer (provided in the kit) at 37°C for 1 h in the dark and then with stop buffer at room temperature for 10 min. After that, the tissue slices were incubated with Streptavidin-HRP solution (provided in the kit) for 30 min and stained with 0.3 ml of DAB buffer (provided in the kit) at room temperature for 20 min. After washing with PBS (M059191, MRREDA) for three times, the images of the tissue slices were observed under an optical microscope (DM4M, Leica, Solms, Germany) at a magnification of ×200.

### Cell Culture and Drug Treatment

Rat cardiomyocytes (H9c2, CRL-1446, ATCC, Manassas, VA, United States) were normally cultured in DMEM (A4192101, Gibco, Waltham, MA, United States) with 10% FBS (16140071, Gibco) at 37°C with 5% CO_2_ in the incubator (HH.CP-T, Grows Instrument Co., Ltd., Shanghai, China), and cell culture medium was changed every 2 days.

The cellular experiment with the H9c2 cell line was performed based on a previous publication (31). For the treatment of paeonol or DOX, the normally cultured H9c2 cells (5 × 10^5^) were first cultured in DMEM with 0.5% FBS for 24 h, and then the cells were cultured with different doses (0.5, 1, and 2.5 mM) of paeonol for 1 h or DOX (5 μM) for 24 h. For the co-treatment of paeonol and DOX, the cells were first treated with paeonol for 1 h and further treated with DOX for 24 h.

### Immunocytochemistry

The H9c2 cells were seeded on plain microscope slides (P3963-01, Aladdin, Shanghai, China) and fixed with 4% paraformaldehyde for 15 min, followed by incubation with 0.1% Triton X-100 (P0096, Beyotime) for 20 min. After the removal of residual liquid, cells were treated with goat serum (C0265, Beyotime) and incubated with primary antibodies against S-phase kinase-associated protein 2 (SKP2) (ab183039, Abcam, United Kingdom) as appropriate. Following the process of washing, the secondary antibodies (ab150077, Abcam) were incubated with the cells, which were finally visualized and observed under a microscope at a magnification of ×200.

### Transfection

miR-21-5p mimic (5′-UAGCUUAUCAGACUGAUGUUGA-3′), mimic control (MC; 5′-UUCUCCGAACGUGUCACGUUU-3′), SKP2 overexpression plasmid, and the empty plasmids (which were used as the negative control) (NC.) were commercially ordered from RIBOBIO (Guangzhou, China).

Prior to cell transfection, H9c2 cells (3.0 × 10^5^) that were cultured in DMEM were added to a six-well plate and maintained until the confluence reached 80%. Then, 2.0 μg of mimic or plasmids was transfected into the cells using 3 μl of lipofectamine 3000 transfection reagent (L3000015, Invitrogen, Waltham, MA, United States) and incubated for 48 h.

### Dual-Luciferase Reporter Assay

Before the commencement of this experiment, the wide-type sequence of SKP2 (SKP2-WT) carrying the binding site of miR-21-5p (5′-AAACTACTTTGAAGAATAAGCTA-3′) and the mutant-type sequence of SKP2 (SKP2-MUT, 5′-AAACTACTTTGAAGAACTAGCAA-3′) with the abrogated binding sites with miR-21-5p were inserted into the pGL3-basic vectors (VT1554, YouBio, Hunan, China), respectively.

For this assay, H9c2 cells (3.0 × 10^4^) grown in the DMEM culture medium were added to a 48-well plate and then co-transfected with the aforementioned vectors of SKP2 and miR-21-5p mimic/mimic control. After the collection of transfected cells, a commercial dual-luciferase reporter assay kit (ab228530, Abcam) was used to process the cells, and the luciferase activities within the cells were finally determined using a SpectraMax reader (Molecular Devices, Shanghai, China).

### RNA Extraction and Reverse-Transcription Quantitative-PCR

Total mRNA was isolated from the heart tissues (100 mg) and cultured in H9c2 cells (1.0 × 10^6^). In brief, the samples were lysed using TRIzol reagent (R0016, Beyotime), mixed with chloroform, and further centrifuged for 20 min (14,000 × *g*). The supernatant was collected and isopropanol was added (H822173, Macklin, Shanghai, China). The total mRNA content was collected following the centrifugation at 14,000 × *g* for 5 min. The miRNA in the heart tissues and cultured H9c2 cells, likewise, were directly extracted using a miRNA isolation regent (DP501, TianGEN, Beijing, China). Then, these RNAs were reverse-transcribed into cDNA by using EasyScript First-Strand cDNA Synthesis SuperMix (AE301-02, TransGen, Beijing, China). After that, the cDNA was amplified using the PerfectStart Green qPCR SuperMix (AQ601-01, TransGen) and the primers of target genes in the instrument of the QuantStudio6 system (Applied Biosystems, CA, United States). The sequences of both forward (F) and reverse (R) primers were as follows: miR-21-5p-F: 5′-TGTTGAGTCGTATCCAGTGCAA-3′, miR-21-5p-R: 5′-GTATCCAGTGCGTGTCGTGG-3′;

U6-F: 5′-CTCGCTTCGGCAGCACATATACTA-3′, U6-R: 5′-ACGAATTTGCGTGTCATCCTTGCG-3′;

SKP2-F: 5′-CTGCAGAATCTGAGTCTGGAAGGC-3′, SKP 2-R: 5′-TAGTGTGGGGATTTCTCCGAGTTC-3′;

GAPDH-F: 5′-CAAGCTCATTTCCTGGTATGAC-3′, GAP DH-R: 5′-CGCTCGAGCAGTCGCTGCAACCATCCA-3′.

It should be noted that for the cells with the treatment of Paeonol or DOX and transfection, the treatment of Paeonol and DOX was performed following the completion of cell transfection.

### Cell Counting Kit-8 Assay

The cardiomyocytes, H9c2, 1.0 × 10^4^) were added to each well of a 96-well plate supplemented with 100 μl of DMEM medium with 10% FBS. After the indicated transfection or treatment with paeonol and DOX, 10 μl of CCK-8 solution (C0040, Beyotime) was added to the 96-well plate and incubated for 4 h. Finally, the 96-well plate was placed in the Imark microplate reader (Bio-Rad, Hercules, CA, United States) to measure the absorbance at 450 nm.

### Flow Cytometry

After the transfection or treatment with paeonol and DOX, the apoptosis of H9c2 cells was detected using an Annexin V-FITC/Propidium iodide (PI) apoptosis detection kit (K2003, APExBIO, Houston, TX, United States) with the use of flow cytometry. In brief, the H9c2 cells (2.0 × 10^5^) were rinsed with PBS twice and incubated with the working solution of Annexin V-FITC and PI at room temperature for 30 min in the dark. Finally, the cells were placed into the FACSCalibur™ flow cytometer (BD Biosciences, Franklin Lake, NJ, United States) to analyze their apoptosis rate.

### 2′,7′-Dichlorodihydrofluorescein Diacetate Staining

After the transfection or treatment with paeonol and DOX, the cardiomyocytes (1.0 × 10^6^) in the six-well plate were washed twice by PBS. The 100 μl of H_2_DCFDA staining buffer (KGAF018, KeyGen Biotech, Nanjing, China) and 100 μl of DMEM were added to each well to stain the cells at 37°C for 60 min. Then, the cells were further cultured in DMEM at 37°C for 30 min. Finally, the images of the cells were observed under a DMI4000B fluorescence microscope (Leica, Solms, Germany) at a magnification of ×200.

### Tetramethylrhodamine Methyl Ester Staining

The cardiomyocytes (1.0 × 10^6^) in the six-well plate which had received the transfection or treatment with paeonol and DOX were first washed by PBS for two times. Then, 200 μl of TMRM staining buffer (T5428, Sigma-Aldrich, St. Louis, MO, United States) was added to each well for staining the cells at 37°C for 30 min. After the stained cells were further washed with PBS twice, the images of the cells were observed under a DMI4000B fluorescence microscope (magnification of ×200, Leica, Solms, Germany).

### Western Blot

The total protein content was extracted from the heart tissues and H9c2 cells (1.0 × 10^6^). In short, NP-40 (P0013F, Beyotime) was used to incubate the samples at room temperature for 15 min and further centrifuged for 20 min (14,000 × *g*) in order to collect the total protein content in the supernatant. After determining its concentration using a BCA protein assay kit (P0009, Beyotime), the total protein (25 μg) was separated in each lane on SDS-PAGE gels (P0052A, Beyotime) and transferred onto PVDF membranes (ISEQ00010, Millipore, MA, United States). The membranes were then blocked by 5% non-fat milk at normal atmospheric temperature for 1.5 h and further incubated with the following primary antibodies at 4°C overnight, including those against SKP2 (1:2,500, 48 kD, ab68455, Abcam, Cambridge, United Kingdom), Bcl-2 (1:2,000, 26 kD, ab196495, Abcam), C-Caspase-3 (1:500, 17 kD, ab49822, Abcam), Bax (1:3,000, 21 kD, ab32503, Abcam), Cyt C (1:500, 12 kD, ab13575, Abcam), GAPDH (1:10,000, 36 kD, ab181602, Abcam), and COXIV (1:500, 17 kD, ab16056, Abcam). The next day, the membranes were incubated with the matching secondary antibody against rabbit IgG (1:5,000, ab205718, Abcam) or the secondary antibody targeting mouse IgG (1:5,000, ab205719, Abcam) at normal atmospheric temperature for 1.5 h. In the end, the membrane was covered with ECL developer solution (P0019, Beyotime), and the protein signal in the membrane was detected and analyzed using the Image Lab 3.0 Software (Bio-Rad, Hercules, CA, United States).

### Statistical Analysis

All the data generated in this study were analyzed by student’s *t*-test and one-way ANOVA. ANOVA was followed by Dunnett’s *post hoc* test/Tukey’s *t*-test. Mean ± standard error of the mean (SEM) was used to indicate the statistical data, which were statistically significant at *P* < 0.05. All analyses were implemented in SPSS 20.0 software.

## Results

### Paeonol Improved the Cardiac Function and Ameliorated the Cardiac Damage of Chronic Heart Failure-Modeled Rats

**FIGURE 1:**
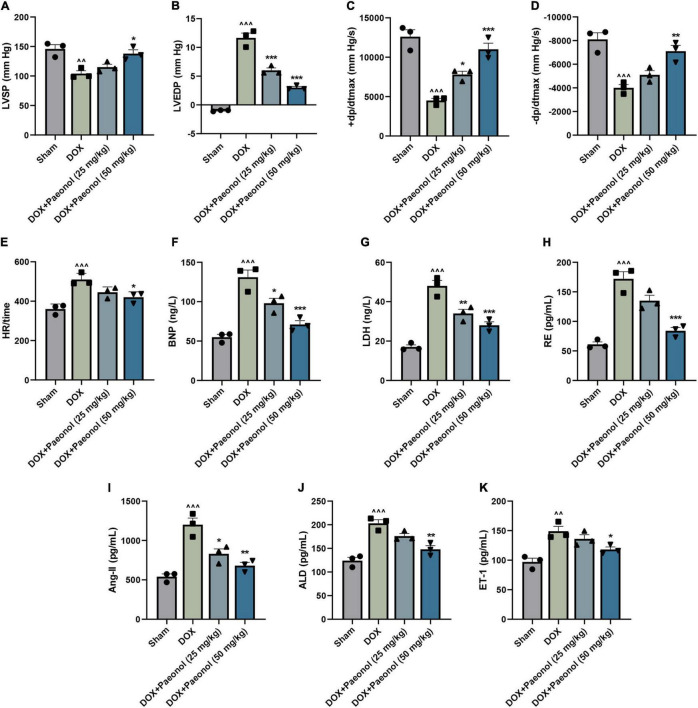
Paeonol improved the cardiac function and ameliorated the cardiac damage of CHF rats. **(A–E)** The parameters underlying the cardiac function of the rats, including LVSP **(A)**, LVEDP **(B)**, +dp/dtmax **(C)**, –dp/dtmax **(D)**, and HR **(E)**, were detected using RM6240 series multichannel physiological signal acquisition apparatus. **(F–K)** The concentration of several specific markers related to the myocardial damage in the rats, including BNP **(F)**, LDH **(G)**, RE **(H)**, Ang II **(I)**, ALD **(J)**, and ET-1 **(K)**, were evaluated using a Synergy H1 Hybrid Multi-Mode Reader. ^∧∧^*P* < 0.01, ^∧∧∧^*P* < 0.001, vs. Sham; *P < 0.05, **P < 0.01, ***P < 0.001, vs. DOX. DOX, doxorubicin; LVSP, left ventricular systolic pressure; LVEDP, left ventricle end diastolic pressure; +dp/dtmax, +left ventricle dp/dtmax; –dp/dtmax, –left ventricle dp/dtmax; HR, heart rate; BNP, brain natriuretic peptide; LDH, lactate dehydrogenase; RE, renin; Ang II, angiotensin II; ALD, aldosterone; ET-1, endothelin 1.

After we established a DOX-induced CHF rat model, the cardiac function of the rats was evaluated (Figures 1A–E), and according to the observations, when compared to the rats in the Sham group, DOX reduced the LVSP (Figure 1A), +dp/dtmax (Figure 1C), and −dp/dtmax (Figure 1D), but increased the LVEDP (Figure 1B) and the HR values (Figure 1E) in the rats (*P* < 0.05). In addition, the value of +dp/dtmax was reduced to 50% of the original value (30), which indicated the successful construction of the CHF model.

After treating the CHF-modeled rats with paeonol, the abnormal hemodynamic parameters of the CHF rat hearts were improved (Figures 1A–E), showing that the cardiac function of CHF rats was improved by paeonol. Furthermore, the cardiac damage of the rats was evaluated by detecting the concentration of several specific markers of myocardial damage (Figures 1E–J). As reflected in the data, the concentrations of the BNP (Figure 1F), LDH (Figure 1G), RE (Figure 1H), Ang II (Figure 1I), ALD (Figure 1J), and ET-1 (Figure 1K) of the CHF-modeled rats were significantly increased when compared to those in the Sham group (*P* < 0.01), while the treatment with paeonol reduced the levels of these markers in these CHF-modeled rats (*P* < 0.05). Collectively, these results demonstrated that paeonol could improve cardiac function and ameliorate the cardiac damage of CHF-modeled rats.

### Paeonol Ameliorated Histopathological Injury, Suppressed the Apoptosis, and Downregulated the Expression of miR-21-5p in the Myocardium of Chronic Heart Failure-Modeled Rats

**FIGURE 2:**
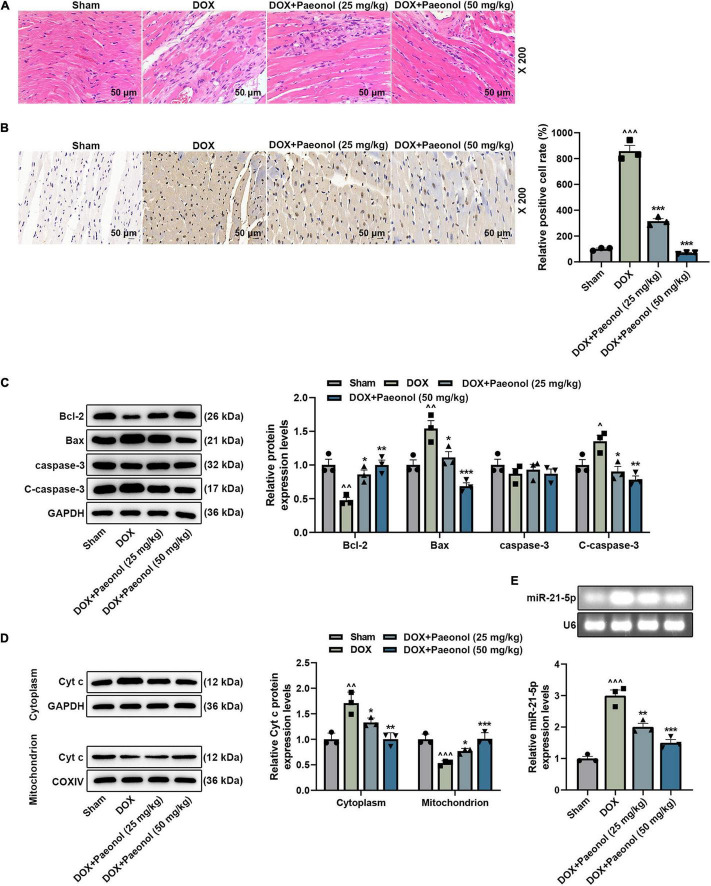
Paeonol ameliorated histopathological injury, suppressed apoptosis, and downregulated the miR-21-5p expression in the myocardium of CHF rats. **(A)** The myocardial histopathology of the rats was detected by hematoxylin–eosin staining (magnification of ×200). **(B)** The myocardial apoptosis of the rats was detected by TUNEL staining (Magnification: ×200). **(C,D)** The levels of Bcl-2, Bax, caspase-3, C-caspase-3, and Cyt C in the myocardium of rats were detected by Western blot. GAPDH was used as an internal control. **(E)** The expression of miR-21-5p in the myocardium of rats was detected by RT-qPCR, with U6 serving as an internal control. ^∧^*P* < 0.05, ^∧∧^*P* < 0.01, ^∧∧∧^*P* < 0.001, vs. Sham; *P < 0.05, **P < 0.01, ***P < 0.001 vs. DOX. DOX, doxorubicin.

To verify our above discoveries, hematoxylin–eosin (Figure 2A) and TUNEL (Figure 2B) staining were performed. As shown in the results (Figure 2A), in the rats of the Sham group, a clear myocardial connective tissue structure and neatly arranged myofibrils in the myocardial tissue were evidenced, and the nuclei were of the same size and shape. However, in the myocardium of rats in the DOX group, however, focal necrosis with myocardial fibrosis, myocardial interstitial edema, and inflammatory cell infiltration were observed. After the treatment with paeonol, the atrophy and degeneration of myocardial fibers, the proliferation of interstitial fibrous tissue, and the significantly reduced number of necrotic cells were observed. In addition, as exhibited in Figure 2B, the apoptosis of the myocardium in the DOX group was significantly increased, but paeonol treatment remarkably inhibited the apoptosis in the myocardium of CHF rats. These phenomena further verified that paeonol improved cardiac function and ameliorated the cardiac damage of CHF rats.

Moreover, we found that DOX inhibited the levels of Bcl-2 and Cyt c (mitochondrion) while promoting the levels of Bax, C-caspase-3, and Cyt c (cytoplasm) in the CHF-modeled rats, but paeonol treatment reversed such effects of DOX (*P* < 0.05; Figures 2C,D). Meanwhile, we also discovered that the expression of miR-21-5p in the CHF-modeled rats was upregulated in comparison to that noticed in the Sham group (*P* < 0.001; Figure 2E), while paeonol treatment remarkably restored the abnormally higher expression of miR-21-5p in the CHF-modeled rats compared to those in the DOX group (*P* < 0.01; Figure 2E), which indicated that miR-21-5p was involved in the mechanism by which paeonol restored the cardiac function of CHF rats.

### Paeonol Increased the Mitochondrial Membrane Potential and Viability but Decreased the Apoptosis, Reactive Oxygen Species, and Downregulated miR-21-5p Expression in the Doxorubicin-Induced Cardiomyocytes

**FIGURE 3:**
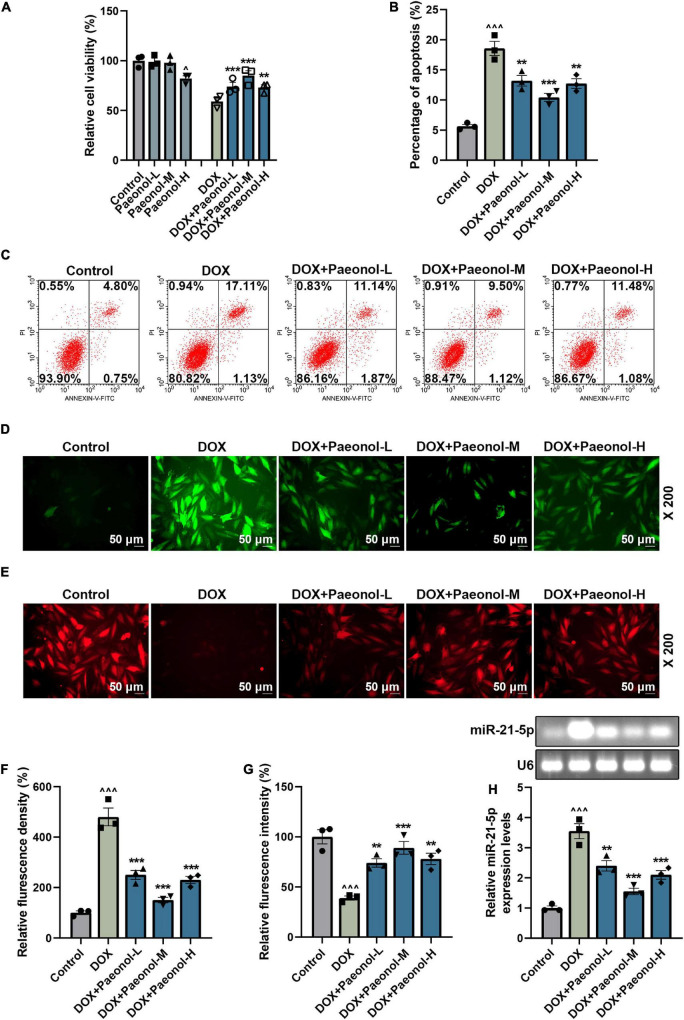
Paeonol increased the MMP and viability, while decreasing the apoptosis and ROS, and suppressing miR-21-5p expression in the DOX-induced cardiomyocytes. **(A)** The viability of H9c2 cells treated with DOX or paeonol was detected by CCK-8 assay. **(B,C)** The apoptosis of H9c2 cells treated with DOX or paeonol was determined *via* flow cytometry. **(D,F)** The level of ROS in the H9c2 cells treated with DOX or paeonol was visualized by H2DCFDA staining. **(E,G)** The MMP in the H9c2 cells received the treatment with DOX or paeonol and was detected using TMRM staining. **(H)** The expression of miR-21-5p in H9c2 cells treated with DOX or paeonol was quantified with the help of RT-qPCR. U6 served as an internal control. ^∧^*P* < 0.05, ^∧∧∧^*P* < 0.001 vs. Control; **P < 0.01, ***P < 0.001 vs. DOX. DOX, doxorubicin; MMP, mitochondrial membrane potential; ROS, reactive oxygen species.

To further analyze the effect of paeonol on CHF, the DOX-induced cardiomyocytes were cultured as appropriate. The results related to the cell viability are presented in Figure 3A, where a low dose of paeonol (Paeonol-L) and a medium dose of paeonol (Paeonol-M) had no effect on the viability of the cardiomyocytes (H9c2), but a high dose of paeonol (Paeonol-H) evidently decreased the viability of the H9c2 cells (*P* < 0.01). In the DOX-induced H9c2 cells, low (Paeonol-L), medium (Paeonol-M), and high (Paeonol-H) doses of paeonol increased the cell viability (*P* < 0.01). As for cell apoptosis (Figures 3B,C), DOX increased the apoptosis in the cardiomyocytes (*P* < 0.001), but paeonol at varied doses decreased the apoptosis of DOX-induced H9c2 cells (*P* < 0.01).

Furthermore, DOX increased the reactive oxygen species (ROS) (Figures 3D,F) and decreased the mitochondrial membrane potential (MMP) (Figures 3E,G) in H9c2 cells (*P* < 0.001), and all these trends were reversed by the administration of varying doses of paeonol (Paeonol-L, Paeonol-M, and Paeonol-H) (*P* < 0.01), which further confirmed that paeonol ameliorated the injury and apoptosis of DOX-induced cardiomyocytes. Moreover, upregulated miR-21-5p was also discovered in the DOX-induced cardiomyocytes (*P* < 0.001; Figure 3H), but all varied doses of paeonol downregulated the expression of miR-21-5p in the DOX-induced cardiomyocytes (*P* < 0.01; Figure 3H). All the discoveries here, in short, proved our findings *in vivo*.

### miR-21-5p Mimic Counteracted the Effect of Paeonol on the Viability, Apoptosis, miR-21-5p Expression, Mitochondrial Membrane Potential, and Reactive Oxygen Species of the Doxorubicin-Induced Cardiomyocytes

**FIGURE 4:**
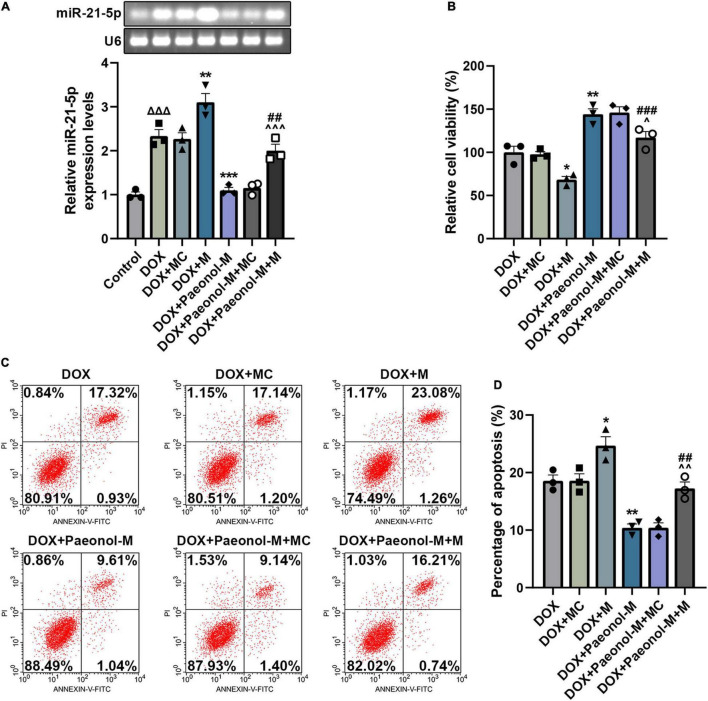
miR-21-5p mimic counteracted the effects of paeonol on the viability, apoptosis, and miR-21-5p expression of the DOX-induced cardiomyocytes. **(A)** The expression of miR-21-5p in DOX-induced H9c2 cells treated with miR-21-5p mimic or paeonol-M was quantified by RT-qPCR. U6 served as an internal control. **(B)** The viability of DOX-induced H9c2 cells treated with miR-21-5p mimic or paeonol-M was detected by CCK-8 assay. **(C,D)** The apoptosis of DOX-induced H9c2 cells treated with miR-21-5p mimic or paeonol-M was unveiled using flow cytometry. *P < 0.05, **P < 0.01, ***P < 0.001 vs. DOX + MC; ^##^*P* < 0.01, ^###^*P* < 0.001, vs. DOX + M;^∧^*P* < 0.05, ^∧∧^*P* < 0.01, ^∧∧∧^*P* < 0.001 vs. DOX + Paeonol-M + MC). DOX, doxorubicin; M, miR-21-5p mimic; MC, mimic control.

To investigate the role of miR-21-5p in the DOX-induced cardiomyocytes, miR-21-5p mimic and paeonol at the medium dosage (abbreviated as Paeonol-M) were further applied. As shown in Figure 4A, the expression of miR-21-5p was upregulated by miR-21-5p mimic (*P* < 0.001) but downregulated by paeonol (*P* < 0.01). After co-treatment with miR-21-5p mimic and paeonol, the effect of paeonol on the expression of miR-21-5p was reversed by the miR-21-5p mimic.

In addition, the viability (Figure 4B) and apoptosis (Figures 4C,D) of the cells were evaluated, and the data showed that miR-21-5p mimic decreased the viability (*P* < 0.05) and induced the apoptosis (*P* < 0.05) of DOX-induced H9c2 cells, while paeonol, on the contrary, increased the viability (*P* < 0.01) and suppressed the apoptosis (*P* < 0.01) of DOX-induced H9c2 cells. After co-treatment with miR-21-5p mimic and paeonol, the effects of paeonol on the viability and apoptosis of DOX-induced cells were reversed by miR-21-5p mimic.

**FIGURE 5:**
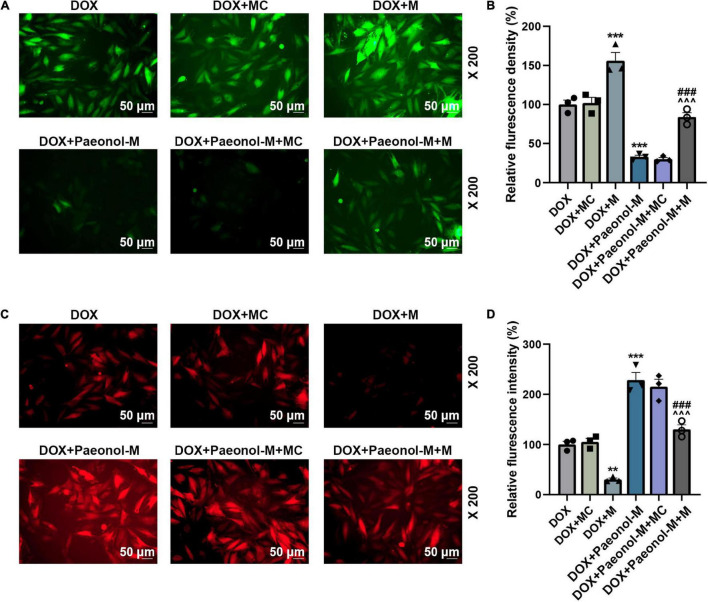
miR-21-5p mimic counteracted the effects of paeonol on the MMP and ROS of the DOX-induced cardiomyocytes. **(A,B)** The ROS level in the DOX-induced H9c2 cells treated with miR-21-5p mimic or Paeonol-M was observed by H2DCFDA staining. **(C,D)** The MMP in the DOX-induced H9c2 cells treated with miR-21-5p mimic or Paeonol-M was determined by TMRM staining. **P < 0.01, ***P < 0.001 vs. DOX + MC;^###^*P* < 0.001, vs. DOX + M;^∧∧∧^*P* < 0.001 vs. DOX + Paeonol-M + MC. DOX, doxorubicin; MMP, mitochondrial membrane potential; ROS, reactive oxygen species; M, miR-21-5p mimic; MC, mimic control.

In addition, the ROS (Figures 5A,B) and MMP (Figures 5C,D) in the cardiomyocytes were determined, and we observed that the ROS level was increased by miR-21-5p mimic (*P* < 0.001) and reduced after the treatment with paeonol (*P* < 0.001), while opposite trends were observed on the MMP after the transfection with miR-21-5p mimic and the treatment with paeonol. Specifically, the MMP in the cardiomyocytes was reduced by miR-21-5p mimic (*P* < 0.01) but was increased following paeonol treatment (*P* < 0.001). After the co-treatment with miR-21-5p mimic and paeonol, the effects of paeonol on the ROS and MMP of DOX-induced cells were neutralized by the miR-21-5p mimic. This evidence thus indicated that the effect of paeonol on DOX-induced cardiomyocytes might be realized through regulating miR-21-5p.

### S-Phase Kinase-Associated Protein 2 Was Targeted by miR-21-5p and Was Downregulated in Chronic Heart Failure-Modeled Rats and Doxorubicin-Induced Cardiomyocytes

**FIGURE 6:**
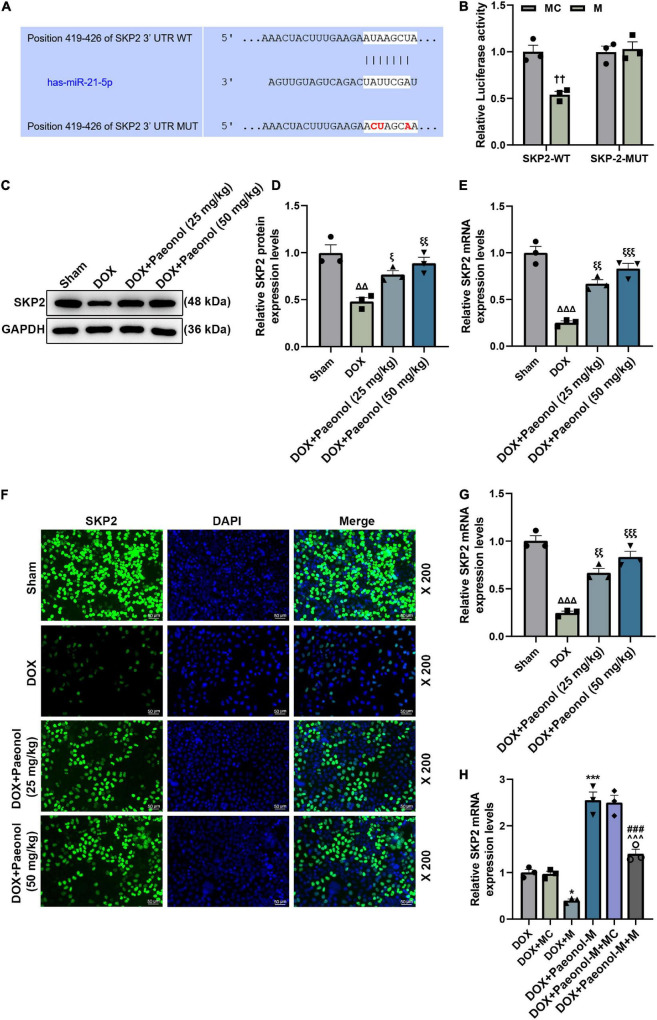
SKP2 was targeted by miR-21-5p and was downregulated in CHF rats and DOX-induced cardiomyocytes. **(A)** The binding sites between miR-21-5p and SKP2 were predicted by the TargetScan database. **(B)** Dual-luciferase reporter assay was employed to verify the targeting relationship between miR-21-5p and SKP2. **(C,D)** The expression of SKP2 in the myocardium of CHF rats was detected by Western blot, with GAPDH serving as an internal control. **(E)** The expression of SKP2 in the myocardium of CHF rats was detected by RT-qPCR. GAPDH was used as an internal control. **(F,G)** The expression of SKP2 in the DOX-induced H9c2 cells treated with PAE was detected by immunocytochemistry. **(H)** The expression of SKP2 in the DOX-induced H9c2 cells treated with miR-21-5p mimic or Paeonol-M was quantified by RT-qPCR. GAPDH served as an internal control. ^††^*P* < 0.01 vs. MC; ^Δ^ ^Δ^ *P* < 0.01, ^Δ^ ^Δ^ ^Δ^ *P* < 0.001 vs. Sham; ^ξ^ *P* < 0.05, ^ξ^ ^ξ^ *P* < 0.01, ^ξ^ ^ξ^ ^ξ^ *P* < 0.001 vs. DOX; *P < 0.05, ***P < 0.001 vs. DOX + MC;^###^*P* < 0.001 vs. DOX + M;^∧∧∧^*P* < 0.001 vs. DOX + PAE-M + MC. DOX, doxorubicin; M, miR-21-5p mimic; MC, mimic control.

Following the prediction of the binding sites between miR-21-5p and SKP2 by the TargetScan database (Figure 6A), the dual-luciferase reporter assay was performed to confirm the targeting relationship between miR-21-5p and SKP2. As shown in Figure 6B, the luciferase activity of the cells co-transfected with SKP2-WT and miR-21-5p mimic was decreased (*P* < 0.01) when compared to those transfected with miR-21-5p mimic control and SKP2-WT, while the insignificant difference in the luciferase activity was identified in the cells co-transfected with SKP2-MUT and miR-21-5p mimic/mimic control, indicating that SKP2 could be targeted by miR-21-5p. Therefore, the expression of SKP2 in the CHF-modeled rats was detected (Figures 6C–E), and the downregulated SKP2 was discovered in these model rats (*P* < 0.01), but the downregulated SKP2 was then restored after paeonol treatment (*P* < 0.05). Similarly, we found that the level of SKP2 in the DOX-induced cardiomyocytes was inhibited, but paeonol upregulated its level in these cardiomyocytes treated with DOX (Figures 6F,G, < 0.01). In addition, the expression of SKP2 in the DOX-induced cardiomyocytes was determined (Figure 6H), with the data revealing that the expression of SKP2 was downregulated by miR-21-5p mimic (*P* < 0.05) and upregulated by paeonol (*P* < 0.001). Additionally, after the co-treatment with the miR-21-5p mimic and paeonol, the effect of paeonol on the expression of SKP2 was reversed by the miR-21-5p mimic. All these phenomena indicated that the effects of paeonol in the CHF-modeled rats and DOX-induced cardiomyocytes might be realized by regulating the miR-21-5p/SKP2 axis.

### S-Phase Kinase-Associated Protein 2 Overexpression Increased the Viability and Mitochondrial Membrane Potential, Decreased the Apoptosis and Reactive Oxygen Species, and Regulated the Apoptosis-Related Factors in miR-21-5p-Treated Doxorubicin-Induced Cardiomyocytes

**FIGURE 7:**
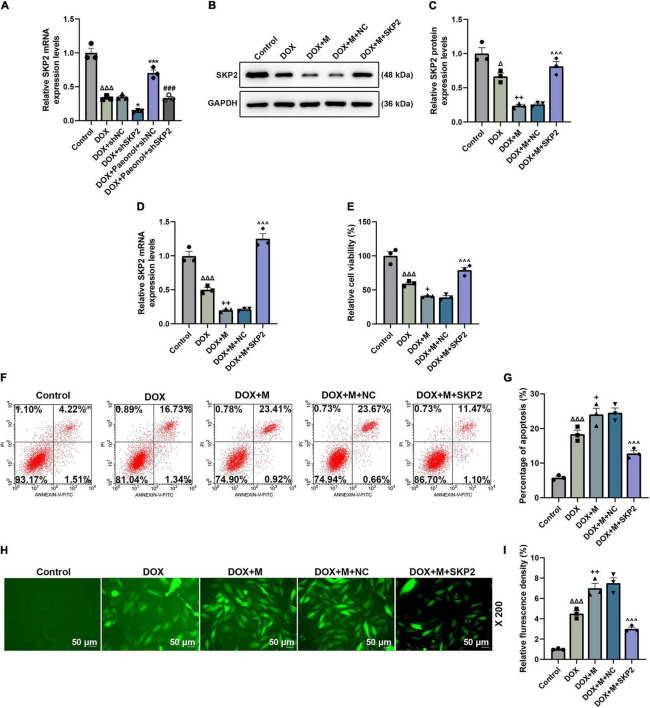
**(A)** The expression of SKP2 in the miR-21-5p-treated DOX-induced H9c2 cells was calculated using RT-qPCR. GAPDH was used as an internal control. **(B,C)** The expression of SKP2 in the miR-21-5p-treated DOX-induced H9c2 cells was detected by western blot, where GAPDH was used as an internal control. **(D)** The levle of SKP2 in the miR-21-5p-treated DOX-induced H9c2 cells was calculated using RT-qPCR. GAPDH was used as an internal control. **(E)** The viability of the miR-21-5p-treated DOX-induced H9c2 cells was evaluated based on CCK-8 assays. **(F,G)** The apoptosis of the miR-21-5p-treated DOX-induced H9c2 cells was determined by flow cytometry. **(H,I)** The ROS of the miR-21-5p-treated DOX-induced H9c2 cells was observable following the H2DCFDA staining (Magnification: ’200). (^Δ^*P* < 0.05, ^ΔΔΔ^*P* < 0.001 vs. Control; *P < 0.05, ***P < 0.001 vs. DOX+shNC; ^###^*P* < 0.001, vs. DOX+Paeonol+shNC; ^+^*P* < 0.05, ^++^*P* < 0.01 vs. DOX; ^∧∧∧^*P* < 0.001, vs. DOX+M+NC). (DOX, doxorubicin; ROS, reactive oxygen species; M, miR-21-5p mimic; NC, negative control).

**FIGURE 8:**
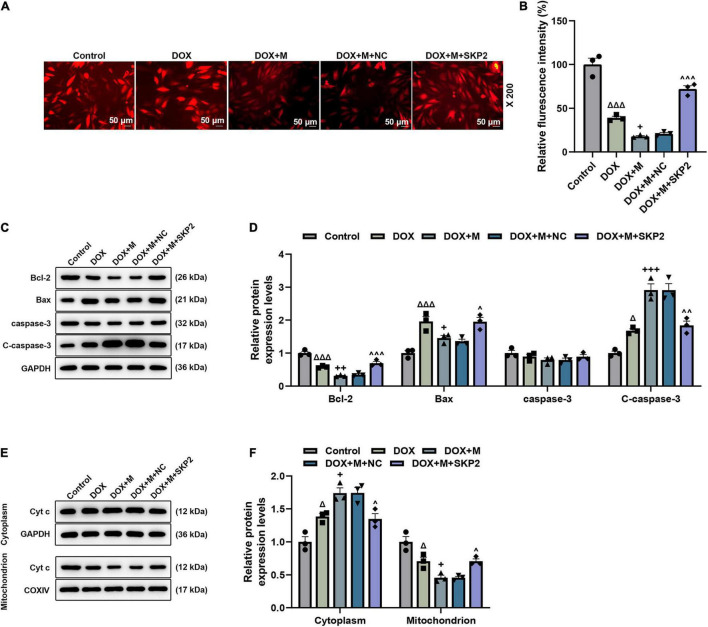
SKP2 overexpression increased the MMP and regulated the apoptosis-related factors in miR-21-5p-treated DOX-induced cardiomyocytes. **(A,B)** The MMP in the miR-21-5p-treated DOX-induced H9c2 cells was determined based on the assay of TMRM staining (Magnification: ×200). **(C,D)** The expression of Bcl-2, Bax, and C-caspase-3 in the miR-21-5p-treated DOX-induced H9c2 cells was calculated by Western blot. GAPDH served as an internal control. **(E,F)** The expression of cytoplasm or mitochondrion Cyt c in the miR-21-5p-treated DOX-induced H9c2 cells was detected *via* Western blot. GAPDH served as an internal control for cytoplasm Cyt c, and COXIV was used as an internal control for mitochondrion Cyt c. ^Δ^ *P* < 0.05, ^Δ^ ^Δ^ ^Δ^ *P* < 0.001 vs. Control;^+^*P* < 0.05, ^++^*P* < 0.01, ^+++^*P* < 0.001 vs. DOX;^∧^*P* < 0.05, ^∧∧^*P* < 0.01, ^∧∧∧^*P* < 0.001 vs. DOX + M + NC. DOX, doxorubicin; MMP, mitochondrial membrane potential; M, miR-21-5p mimic; NC, negative control.

The role of SKP2 in the DOX-induced cardiomyocytes that were transfected with miR-21-5p mimic or SKP2 overexpression plasmid was analyzed. The translation (Figures 7A,B) and transcription (Figure 7C) levels of SKP2 in the miR-21-5p-treated DOX-induced H9c2 cells were promoted by SKP2 overexpression (*P* < 0.05). The viability of the DOX-induced H9c2 cells that had been transfected with miR-21-5p mimic was also increased by overexpressed SKP2 (*P* < 0.001; Figure 7D), while the apoptosis (Figures 7E,F) and the ROS (Figures 7G,H) of the miR-21-5p-treated DOX-induced H9c2 cells were decreased following SKP2 overexpression (*P* < 0.05). In addition, SKP2 overexpression increased MMP of the miR-21-5p-treated DOX-induced H9c2 cells (*P* < 0.05; Figures 8A,B). All the above-mentioned findings indicated that the effects of miR-21-5p on the viability and apoptosis of DOX-induced cells were realized by regulating SKP2.

To further verify this discovery at a molecular level, the expression of apoptosis-related factors was analyzed. After the overexpression of SKP2, the expression of Bcl-2 and C-caspase-3 was upregulated (*P* < 0.05; Figures 8C,D), while the expression of Bax (*P* < 0.05; Figures 8C,D) was downregulated. Furthermore, following the overexpression of SKP2, the expression of Cyt c in the cytoplasm was downregulated (*P* < 0.05; Figures 8E,F) but that in the mitochondrion (*P* < 0.05; Figures 8E,F) was upregulated. These data not only verified our above findings but also demonstrated that SKP2 overexpression reduced the apoptosis of DOX-induced H9c2 cells which had been previously induced with the overexpression of miR-21-5p.

## Discussion

The induction of DOX or isoproterenol, constriction of the abdominal aorta, and ligation of the coronary artery have been proposed to be applied in the establishment of animal HF models (28, 30). The method of DOX induction is easy to be implemented, with low mortality and a high success rate of modeling, and it can also predict the time when the symptoms of CHF will appear (29). Therefore, the method of intraperitoneal injection of DOX is used to establish a rat model of CHF in this study, which turns out to be successful, as reflected by the discovery of the abnormal indexes of cardiac function and the reduced value of +dp/dtmax to 50% of the original value. What should be also noted in our current research on the efficacy of paeonol in CHF is that the treatment with paeonol ameliorated the abnormalities of these indexes, indicating that paeonol might exert a therapeutic effect on CHF. There are also serum-specific markers of myocardial damage, including the factors in the Renin angiotensin–aldosterone system (RAAS), such as BNP, LDH, RE, Ang-II, ALD, and ET-1 (28). The increasing levels of these factors are indicative of CHF occurrence. In this study, we discovered that paeonol treatment inhibited these increasing factors and mitigated the histopathological injury and the apoptosis of myocardiocytes in CHF rats, which proved that paeonol indeed exerted a therapeutic effect on CHF, although the mechanism underlying these effects required further exploration.

The previous study has proved that the downregulation of miR-21 suppresses the cardiac alterations induced by DOX (26). Furthermore, the anti-tumor effect of paeonol on liver cancer could be realized by regulating the miR-21-5p axis, a mature body of miR-21 (27). Based on these findings, we speculated whether paeonol could affect CHF by regulating miR-21-5p. Interestingly, the expression of miR-21-5p was found to be upregulated in the CHF-modeled rats, which was further restored to normal by paeonol treatment, which indicated that the effect of paeonol on CHF might be realized by regulating miR-21-5p. For further verification, the model of DOX-induced cardiomyocytes was established, and these cardiomyocytes were processed with paeonol and miR-21-5p mimic. The experimental data revealed that DOX decreased the viability and increased the apoptosis of cardiomyocytes, but paeonol increased the viability and inhibited the apoptosis of DOX-induced cardiomyocytes. The effects of miR-21-5p were contrary to those of paeonol, and what is more, miR-21-5p further reversed the effects of paeonol, which shows that paeonol regulated miR-21-5p to mitigate the DOX-induced injury on cardiomyocytes. The injury of cardiomyocytes during CHF is mainly caused by oxidative stress and disorder in the energy metabolism of myocardial cells (28, 32). The ROS is generated, and excessive accumulation of ROS will lead to tissue damage under oxidative stress (33). During myocardial energy metabolism disorder, the myocardial energy is reduced, and the transition pores in the mitochondrial membrane of myocardial cells are over-opened, thus increasing the permeability of the membrane and causing damage to mitochondria (32, 33). Both ROS accumulation and mitochondrial damage can cause the loss of MMP (32). In this study, paeonol decreased the ROS and promoted MMP of the DOX-induced cardiomyocytes, while miR-21-5p mimic showed the opposite results and further reversed the effects of paeonol. These findings thus proved that paeonol mitigated the DOX-induced injury on cardiomyocytes by regulating the miR-21-5p axis.

To further explore the mechanism underlying the effects of paeonol and miR-21-5p in CHF, we then determined the potential target gene of miR-21-5p. Based on the prediction of bioinformatics and the confirmation *via* dual-luciferase reporter assay, SKP2 was found to be targeted by miR-21-5p. SKP2, a member of the F-box family, plays an important role in the modulation of the cell cycle (34–36). It is also reported that SKP2 has an anti-tumor effect on various types of cancers, such as breast cancer, lung cancer, osteosarcoma, and so on (37–40). Furthermore, SKP2 expression could improve post-ischemic cardiac function, and it has a therapeutic effect on pathological cardiac hypertrophy (41, 42). In this study, for the first time, the downregulated SKP2 level was evidenced in both CHF rats and DOX-induced cardiomyocytes, in addition to the suggestion that the expression of SKP2 was promoted by paeonol but was inhibited by miR-21-5p mimic. What should be additionally noted is that SKP2 overexpression reversed the effects of miR-21-5p mimic on the viability, apoptosis, MMP, ROS, and the expression of apoptosis-related factors in DOX-induced cardiomyocytes. In brief, all these phenomena suggested that the effects of miR-21-5p on the DOX-induced injury of cardiomyocytes were mediated by targeting SKP2.

Collectively speaking, the present study revealed that paeonol exerts a therapeutic effect on CHF, and in detail, its cardioprotective effect on CHF is confirmed by downregulating miR-21-5p, a miRNA that further targets SKP2. However, it is not clear whether the administration of paeonol prior to that of DOX causes myocardial injury in rats, which is a limitation of this study. In short, it is suggested that paeonol, miR-21-5p, and SKP2 may serve as novel candidates for the diagnosis or treatment of CHF.

## Data Availability Statement

The original contributions presented in this study are included in the article/Supplementary Material, further inquiries can be directed to the corresponding author.

## Ethics Statement

The animal study was reviewed and approved by Animal trials in this study were approved by the Committee of Experimental Animals of the University of Hong Kong Shenzhen Hospital (Z2020011901X) and performed in the University of Hong Kong Shenzhen Hospital.

## Author Contributions

CC had made a substantial contributions to conception and design and drafting the article or critically revising it for important intellectual content. SL, GC, YH, RW, MW, ML, and QY contributed to the data acquisition, data analysis, and interpretation. All authors agreed to be accountable for all aspects of the work in ensuring that questions related to the accuracy or integrity of the work are appropriately investigated and resolved. All authors had approved the final version to be published.

## Conflict of Interest

The authors declare that the research was conducted in the absence of any commercial or financial relationships that could be construed as a potential conflict of interest.

## Publisher’s Note

All claims expressed in this article are solely those of the authors and do not necessarily represent those of their affiliated organizations, or those of the publisher, the editors and the reviewers. Any product that may be evaluated in this article, or claim that may be made by its manufacturer, is not guaranteed or endorsed by the publisher.
