# Supplementary material for: Cardioprotective Effect of Paeonol on Chronic Heart Failure Induced by Doxorubicin via Regulating the miR-21-5p/S-Phase Kinase-Associated Protein 2 Axis
Source: Front Cardiovasc Med. 2022 Jul 5;9:695004. doi: 10.3389/fcvm.2022.695004 (PMC9294229; doi:10.3389/fcvm.2022.695004)

Figure 2C

Bcl-2

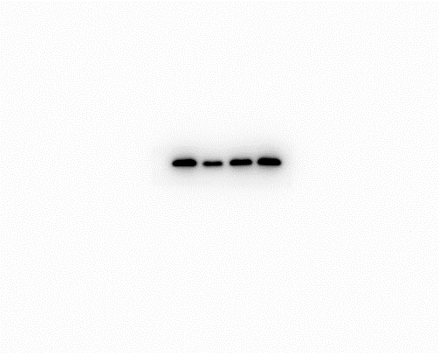

Bax

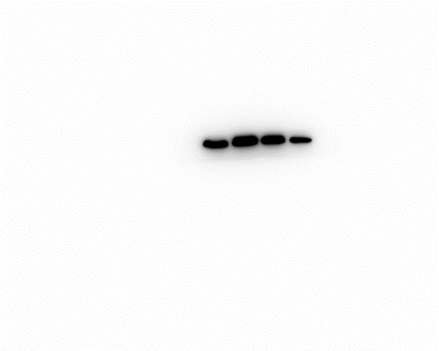

caspase-3

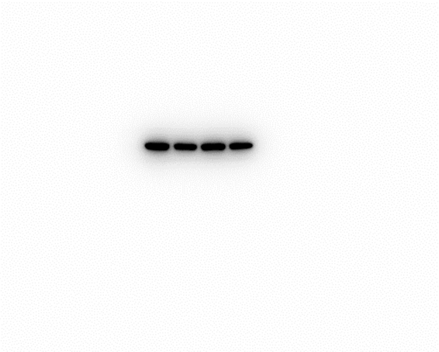

C-caspase-3

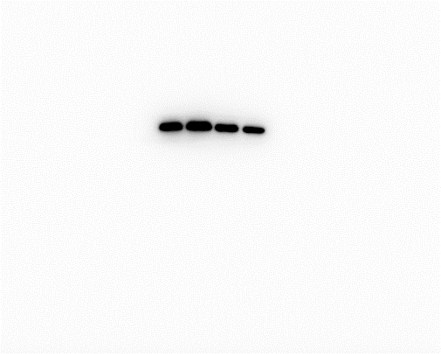

GAPDH

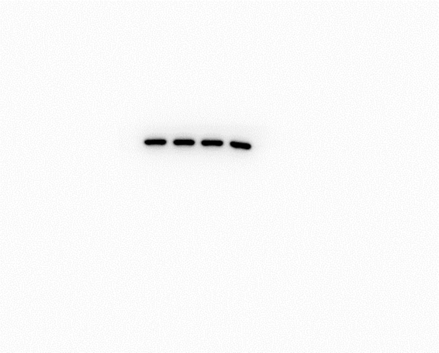

Figure 2D

Cyt c

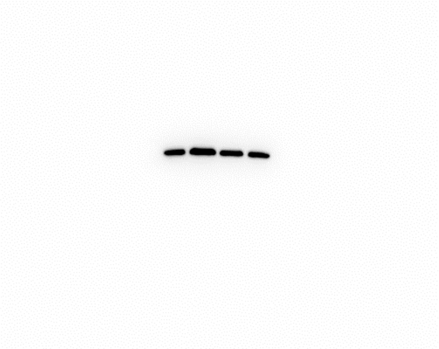

GAPDH

Cyt c

COXIV

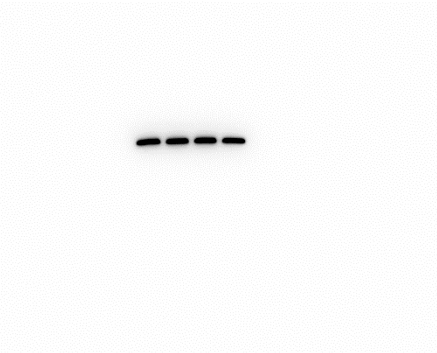

Figure 6C

SKP2

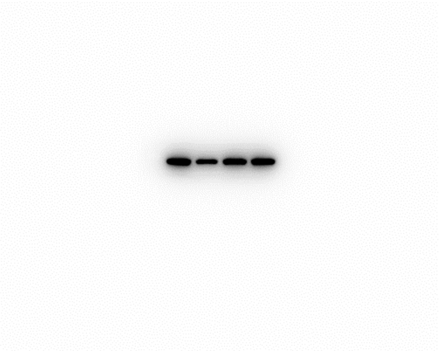

GAPDH

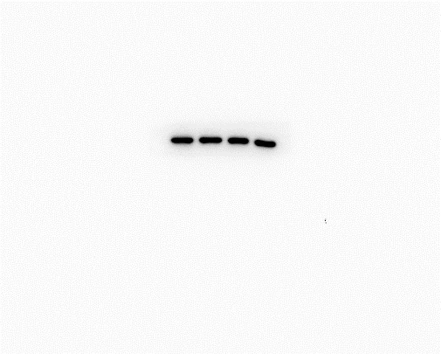

Figure 7B

SKP2

GAPDH

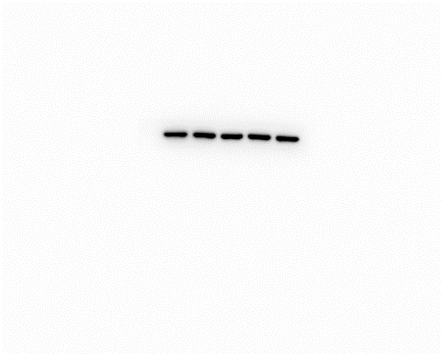

Figure 8C

Bcl-2

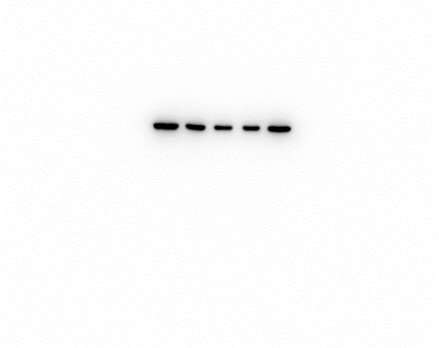

Bax

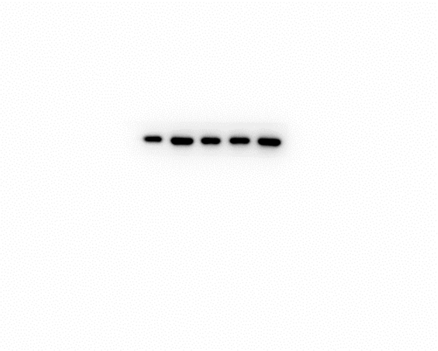

caspase-3

C-caspase-3

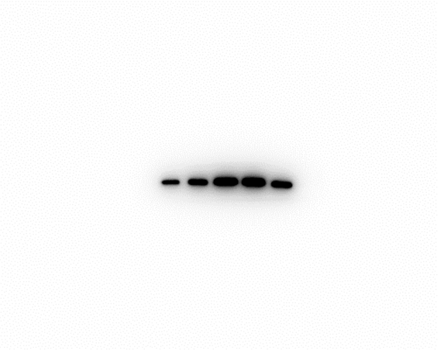

GAPDH

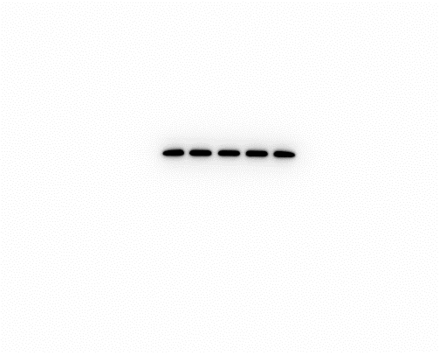

Figure 8E

Cyt c

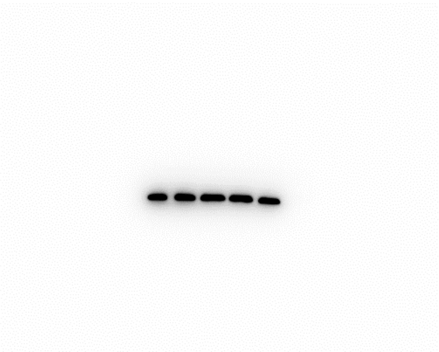

GAPDH

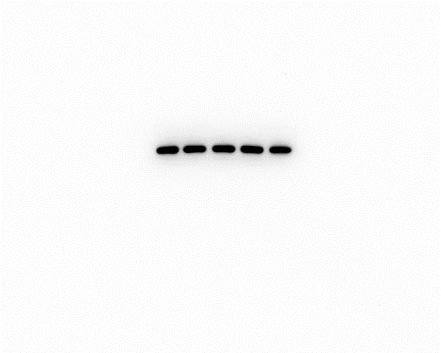

Cyt c

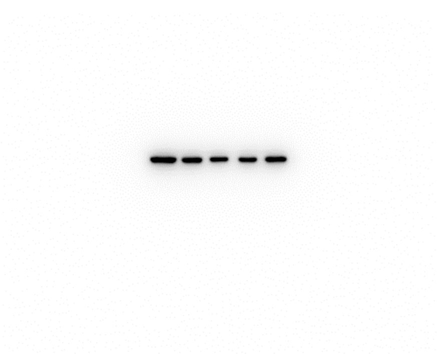

COXIV

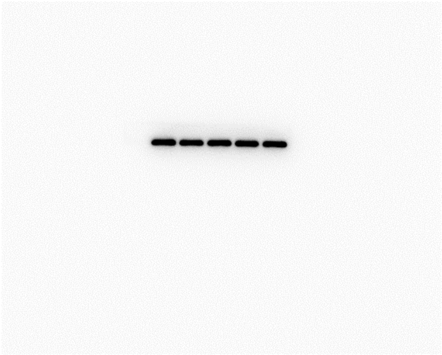

Supplement: Supplementary file 1 [file Data_Sheet_1.PDF]
