# Supplementary figures and images for: Cardioprotective Effect of Paeonol on Chronic Heart Failure Induced by Doxorubicin via Regulating the miR-21-5p/S-Phase Kinase-Associated Protein 2 Axis
Source: Front Cardiovasc Med. 2022 Jul 5;9:695004. doi: 10.3389/fcvm.2022.695004 (PMC9294229; doi:10.3389/fcvm.2022.695004)

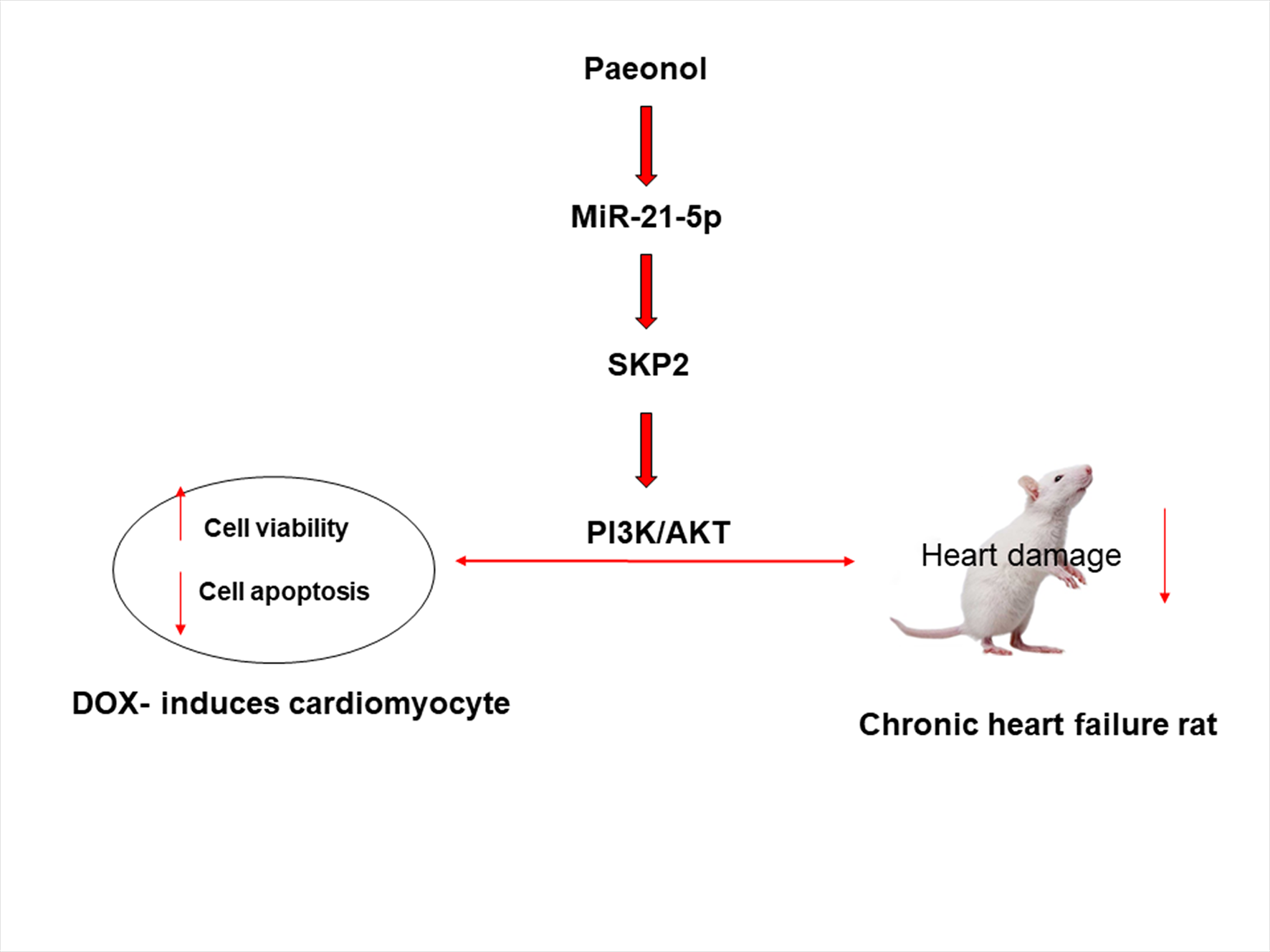

Supplement: Supplementary file 2 [file Image_1.TIF]
